# Performance of Large Language Models Across Diagnostic Tasks in Oral and Maxillofacial Pathology: A Systematic Review

**DOI:** 10.1159/pat/aehag008

**Published:** 2026-10-16

**Authors:** Sohaib Shujaat, Marryam Riaz, Hisham Albrikeet, Hongyang Ma

**Affiliations:** a King Abdullah International Medical Research Center, Department of Maxillofacial Surgery and Diagnostic Sciences, College of Dentistry, King Saud Bin Abdulaziz University for Health Sciences, Ministry of National Guard Health Affairs, Riyadh, Kingdom of Saudi Arabia; b Department of Physiology, Azra Naheed Dental College, Superior University, Lahore, Pakistan; c King Abdullah International Medical Research Center, College of Dentistry, King Saud Bin Abdulaziz University for Health Sciences, Ministry of National Guard Health Affairs, Riyadh, Kingdom of Saudi Arabia; d Department of Oral Implantology, The Second Affiliated Hospital of Harbin Medical University, Harbin, China

**Keywords:** Oral and maxillofacial pathology, Large language models, Artificial intelligence, Diagnostic accuracy, Machine learning, Oral squamous cell carcinoma

## Abstract

**Introduction:**

Large language models (LLMs) are increasingly evaluated for diagnostic applications; however, studies in oral and maxillofacial pathology (OMFP) assess materially different diagnostic tasks and outcomes. This systematic review synthesized evidence according to the specific task evaluated.

**Methods:**

PubMed, Web of Science, and Cochrane Central were searched without language or date restrictions, supplemented by gray-literature screening and citation tracking. Studies evaluating defined OMFP diagnostic tasks using task-appropriate outcomes were included. Screening, data extraction, and quality assessment were independently performed by 2 reviewers. Owing to clinical and methodological heterogeneity, evidence was synthesized narratively by diagnostic task.

**Results:**

Sixteen studies published between 2024 and 2025 were included. Seven evaluated single-diagnosis identification, 6 differential-diagnosis generation or ranking, 5 binary or multiclass classification/detection, and 1 structured diagnostic report generation; counts were non-mutually exclusive. Exact-diagnosis performance varied considerably and did not demonstrate consistent specialist equivalence. Differential-diagnosis studies generally reported higher Top-k inclusion than Top-1 accuracy, but these outcomes did not establish exact diagnostic correctness. Classification studies frequently reported high specificity but variable sensitivity. The single report-generation study reported item-level rather than whole-case diagnostic outcomes.

**Conclusion:**

Large language models may support selected OMFP tasks, particularly differential-diagnosis generation. However, exact-diagnosis performance remains inconsistent, classification sensitivity is task-dependent, and evidence for structured report generation is limited. Current evidence does not support a single overall estimate of diagnostic performance or autonomous diagnostic use.

## Introduction

Oral and maxillofacial pathology (OMFP) encompasses a broad spectrum of mucosal and osseous diseases, including inflammatory, infectious, potentially malignant, and malignant conditions [1, 2]. Accurate and timely diagnosis is essential for appropriate clinical management, prognostication, and prevention of disease progression or malignant transformation [3, 4]. However, diagnostic accuracy in OMFP can vary substantially among clinicians due to overlapping clinical and radiographic features, variability in image quality, limited access to specialist oral pathology services, and continued reliance on invasive biopsy as the diagnostic gold standard [5, 6]. These challenges underscore the need for reliable adjunctive decision-support systems capable of enhancing diagnostic reasoning and improving triage accuracy across diverse clinical settings [7, 8].

Artificial intelligence (AI) has shown increasing promise in supporting diagnostic processes within pathology. Conventional deep learning approaches, particularly convolutional neural networks (CNNs), have demonstrated high performance in image-based tasks such as segmentation, lesion detection, and classification [9, 10]. In oral medicine and pathology, CNN-based models have been investigated for identifying oral potentially malignant disorders and differentiating benign from malignant mucosal lesions [11]. Despite their demonstrated accuracy, these models are typically task-specific, require large annotated datasets, and lack the capacity for contextual reasoning. They often perform well in narrowly defined image-based tasks but have limited ability to integrate clinical context, articulate diagnostic uncertainty, or provide explanatory reasoning, features that are fundamental to diagnostic pathology practice [12, 13].

Large language models (LLMs) represent an emerging paradigm in medical AI [14]. Unlike traditional image classifiers, LLMs are trained on extensive and heterogeneous datasets that include textual information and, in some architectures, visual data [15]. These models are capable of interpreting unstructured clinical narratives, structured pathology reports, and, in multimodal configurations, clinical or radiographic images [16, 17]. Through advanced prompting strategies such as few-shot learning, retrieval-augmented generation, and chain-of-thought reasoning, LLMs can generate diagnostic hypotheses, propose differential diagnoses, and produce context-aware explanations [18–20]. This capacity for multimodal reasoning parallels the integrative diagnostic workflow employed by oral and maxillofacial pathologists, who synthesize clinical, radiographic, and histopathologic information to arrive at a final diagnosis [21, 22].

Large language models are increasingly being explored across multiple medical specialties for diagnostic and decision-support applications [23, 24]. In the context of OMFP, however, their diagnostic role remains incompletely characterized [25]. Early studies have investigated the application of LLMs to oral diagnostic tasks such as lesion interpretation, analysis of clinical narratives, and classification of histopathologic text [26]. Nevertheless, the available evidence remains fragmented, and no systematic review has yet synthesized LLM evaluations across distinct diagnostic tasks in OMFP. Existing reviews in dental and oral AI have largely focused on traditional machine learning and deep learning approaches [27, 28], leaving generative and reasoning-based models relatively underexplored.

The primary objective of this systematic review was to evaluate and synthesize evidence on distinct diagnostic tasks performed by LLMs in OMFP. Specifically, this review aims to distinguish the diagnostic tasks evaluated by LLMs; examine the influence of text-only, image-only, and multimodal inputs within each task; assess task-specific outcomes against the reported reference standards and comparators; and identify methodological limitations and research gaps relevant to future validation and clinical integration.

## Methods

### Protocol and Registration

This systematic review was conducted following the Preferred Reporting Items for Systematic Reviews and Meta-Analyses of Diagnostic Test Accuracy Studies (PRISMA-DTA) guidelines [29]. The protocol was registered with the PROSPERO (Prospective Register of Systematic Reviews) database under the registration number CRD420251184937. As no primary human or animal data were collected, institutional review board approval and informed consent were not required. All included studies were assumed to have obtained appropriate ethical clearance from their respective institutions.

### Review Question

The research question was formulated using the PICO (Population, Intervention, Comparator, and Outcome) framework:

*Population (P):* Human participants or clinical cases involving OMFP.

*Intervention (I):* Use of an LLM to perform a defined OMFP diagnostic task.

*Comparator (C):* Study-defined reference standard used to determine correctness, such as histopathological confirmation, expert consensus, or the confirmed diagnosis reported in the source case. Human clinicians, other LLMs, and conventional AI systems were recorded separately as performance comparators where available and were not treated as reference standards.

*Outcome (O):* Outcomes appropriate to the diagnostic task evaluated: exact-match accuracy for single-diagnosis identification; Top-1 ranking accuracy, Top-k inclusion, or other ranking measures for differential-diagnosis generation; accuracy, sensitivity, specificity, F1-score, and area under the curve (AUC) for defined classification or detection tasks; and item-level accuracy and recall for structured diagnostic report generation. Agreement, clinical plausibility, calibration, report quality, and repeatability were treated as ancillary output characteristics rather than measures of diagnostic correctness.

“ In oral and maxillofacial pathology, what task-specific performance have LLMs demonstrated in single-diagnosis identification, differential-diagnosis generation and ranking, binary or multiclass lesion classification/detection, and structured diagnostic report generation, when evaluated against the study-defined reference standard and, where available, human comparators?”

### Eligibility Criteria

This systematic review included original research articles that evaluated LLMs in one or more defined OMFP diagnostic tasks: single-diagnosis identification, differential-diagnosis generation or ranking, binary or multiclass classification/detection, or structured diagnostic report generation. Eligible studies specifically addressed diseases or lesions involving the oral cavity, jaws, maxillofacial bones, salivary glands, or associated mucosal and soft-tissue structures. Text-based, image-based, and multimodal diagnostic applications of LLMs were considered. To be included, studies were required to report measurable outcomes appropriate to the diagnostic task being evaluated.

Studies were excluded if they focused primarily on educational or theoretical applications (e.g., multiple-choice question answering or simulated oral pathology teaching), nondiagnostic uses (such as patient communication, education, or awareness tools), multiple-choice or examination-style question answering with fixed answer options, or studies focused exclusively on non-oral head-and-neck regions, including the pharynx, larynx, esophagus, thyroid gland, and general head and neck oncology. Mixed-site studies were eligible when they included directly relevant oral-lesion data and the diagnostic task was applicable to OMFP. Reviews, commentaries, editorials, questionnaires, surveys, conference abstracts, and non-peer-reviewed materials were also excluded. No language or publication date restrictions were applied during the selection process to ensure comprehensive coverage of relevant studies.

### Information Sources and Search Strategy

An electronic search was performed across PubMed, Web of Science, and the Cochrane central databases, without restrictions on language or publication year, up to November 2025.

A comprehensive search strategy was developed using a combination of MeSH terms and free-text keywords related to OMFP and LLM-based systems. The core search terms covered concepts such as “oral and maxillofacial pathology,” “oral lesions,” “potentially malignant disorders,” “oral cancer,” “generative artificial intelligence,” and “large language models.” Database-specific search strings and the PRISMA 2020 checklist are provided as online supplementary Files 1 and 2 (for all online suppl. material, see https://doi.org/10.1159/pat/aehag008), respectively.

A gray-literature search was conducted using Google Scholar and OpenGrey, and additional studies were identified through manual screening of reference lists and citation tracking of included articles. All identified records were imported into EndNote 2025 (Clarivate, Philadelphia, PA, United States of America) for duplicate removal and screening.

### Study Selection and Data Extraction

Two reviewers (H.A. and M.R.) independently screened the titles and abstracts of all retrieved studies. Full-text articles were then assessed for eligibility according to the inclusion and exclusion criteria. Any disagreements were resolved through discussion, and a third reviewer (S.S.) was consulted when consensus could not be achieved. Data extraction was independently performed by the same 2 reviewers using the standardized data-extraction-form, with discrepancies resolved through discussion and, when necessary, third-reviewer adjudication.

A standardized data-extraction table was developed to ensure consistency across studies. Extracted variables included bibliographic details, study design, diagnostic domain, diagnostic task, target output, lesion category, data source, input modality, sample size, LLM name and version, model type, comparator, reference standard, and outcome metrics. Each study’s key findings were also summarized. Input modality (text-only, image-only, or multimodal) was extracted as a secondary descriptive variable because of its potential influence on task-specific outcomes and clinical applicability. Lesion categories (e.g., inflammatory/immune-mediated lesions, oral potentially malignant disorders, malignant epithelial lesions, benign/reactive lesions, and mixed OMFP entities) were also recorded to facilitate secondary interpretation within each diagnostic-task group. When necessary, corresponding authors were contacted to obtain missing data or clarifications. For each study, the task-specific outcome measure, reference standard, and comparator were extracted and interpreted separately; agreement, plausibility, report quality, and repeatability were not treated as measures of diagnostic correctness.

### Data Synthesis and Heterogeneity Strategy

Clinical and methodological heterogeneity was anticipated a priori due to variability in input modality, diagnostic task design, lesion spectrum, reference standards, and outcome reporting across eligible studies. Heterogeneity was expected across the following domains: (1) input modality (text-only, image-only, multimodal); (2) diagnostic task type (single-diagnosis identification, differential-diagnosis generation or ranking, binary or multiclass classification/detection, or structured diagnostic report generation); (3) lesion spectrum (inflammatory/immune-mediated, potentially malignant disorders, malignant epithelial lesions, mixed lesions, and histopathology-based text interpretation); (4) reference standards (histopathology, specialist consensus, or published case-report diagnosis); (5) comparator types (clinicians, CNN baselines, or within-model comparisons); and (6) task-specific outcome measures, including exact-match accuracy for single-diagnosis identification; Top-1 ranking accuracy, Top-k inclusion, or other ranking measures for differential diagnosis; accuracy, sensitivity, specificity, F1-score and AUC for classification or detection; and item-level accuracy or recall for structured report generation. Agreement, plausibility, calibration and repeatability were considered separately as ancillary output characteristics.

Given that these constructs are not directly comparable, statistical pooling and meta-analysis were not performed. Instead, a structured narrative synthesis was undertaken using diagnostic task type as the primary analytical axis. Within each diagnostic-task group, the target output, task-appropriate outcome, reference standard, and comparator were reported explicitly. Input modality and lesion category were interpreted as secondary sources of heterogeneity.

### Risk-of-Bias Assessment

The Quality Assessment of Diagnostic Accuracy Studies-2 (QUADAS-2) tool was used to evaluate the risk of bias and applicability of included studies. Four domains were assessed:

*Patient selection:* Appropriateness of inclusion criteria, avoidance of case–control design, and representativeness of samples.

*Index test:* Blinding of LLM assessment from reference standard results and independence of interpretation.

*Reference standard:* Adequacy and validity of the gold standard diagnosis and its independence from the index test.

*Flow and timing:* Consistency of data inclusion and appropriate interval between LLM evaluation and reference standard determination.

Each domain was judged for risk of bias (low, high, unclear), and the first three were also assessed for concerns regarding applicability. Two reviewers conducted the assessment independently, with discrepancies resolved by consensus or third-reviewer arbitration.

## Results

### Study Selection

The study selection process is illustrated in the PRISMA flow diagram (Fig. 1). A total of 311 records were identified through electronic database searching and gray-literature screening. After removal of 61 duplicates, 250 unique records underwent title and abstract screening. Of these, 231 were excluded for failing to meet the inclusion criteria. Nineteen full-text articles were assessed for eligibility, and three were excluded following full-text evaluation. Sixteen studies met all eligibility criteria and were included in the qualitative synthesis [30–45].

**Fig. 1.**
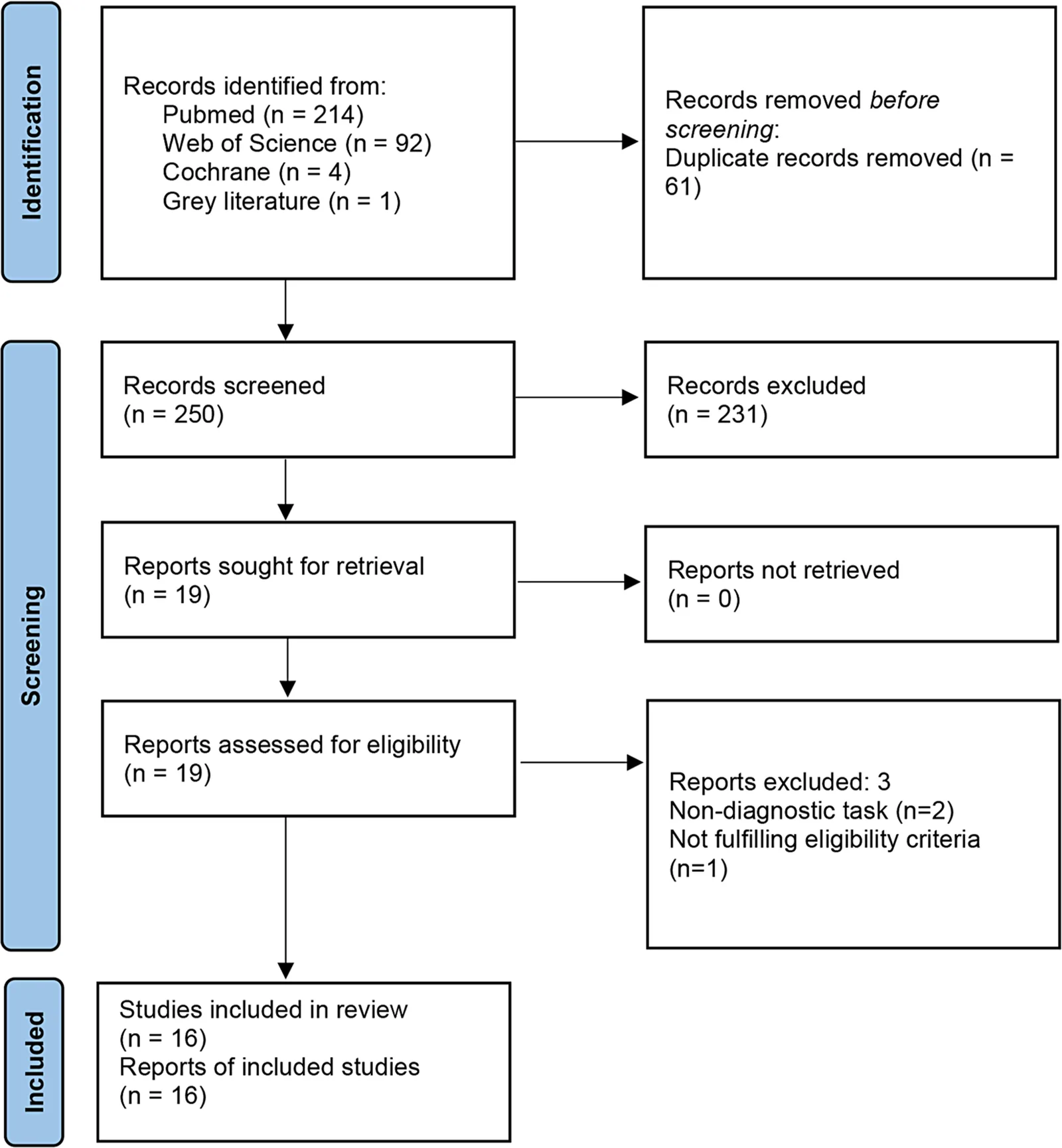
PRISMA flow diagram illustrating the identification, screening, eligibility assessment, and inclusion of studies in the systematic review.

### Study Characteristics and Sources of Heterogeneity

Table 1 summarizes the characteristics of the included studies. Sixteen studies published between 2024 and 2025 were included. All evaluated one or more LLM-based diagnostic tasks in OMFP, although the clinical focus, target output, study design, reference standard, and evaluation framework varied considerably. Diagnostic domains included oral potentially malignant disorders and mucosal lesions [34, 37, 43], oral squamous cell carcinoma and leukoplakia [32, 44], oral lichen planus (OLP) [31, 42, 44], mixed benign, premalignant and malignant lesions [39, 45], challenging maxillofacial pathologies [41], and histopathologic text-based diagnostic interpretation [36].

**Table 1.** Study Characteristics

Authors	Year	Country	Study design/evaluation type	Domain	Task	Data source	Input modality	Sample size	LLM(s) used	Comparator	Reference standard
Tomo et al. [30]	2024	Brazil	Comparative diagnostic performance	Oral and maxillofacial diseases (oral lesions, salivary gland and jaw lesions)	Differential diagnosis	Specialist-confirmed clinical cases plus additional dentist-described cases	Text-only clinical descriptions	37 cases	ChatGPT-3.5, ChatGPT-4	Human experts (oral and maxillofacial pathologists *n* = 18; general dentists *n* = 23; dental students *n* = 16)	Final clinical diagnosis (biopsy/imaging when available)
Yu et al. [31]	2024	China	Diagnostic performance	OLP	Single-diagnosis OLP recognition	Clinically diagnosed OLP intraoral photographs from a hospital cohort, independently reviewed by 3 oral mucosal disease specialists	Image-only (intraoral photos)	128 images	ChatGPT-4o, ChatGPT with Chat-Diagrams plug-in, Claude Opus	None	3 oral mucosal disease specialists using diagnostic guidelines and ICD-11
Schmidl et al. [32]	2025	Germany	Exploratory diagnostic accuracy	Oral/oropharyngeal squamous cell carcinoma, leukoplakia, and non-lesion images	Three-class classification (SCC/leukoplakia/no lesion)	Hospital clinical images with paired standardized clinical history fields	Image-only/Text-only/Image+history	45 images	ChatGPT-4.0	None	Histologically confirmed pathological cases; no-lesion images served as controls
Zhan et al. [33]	2025	China	Diagnostic performance	Oral mucosal diseases	Structured diagnostic report generation	60 textbook mucosal disease cases + 60 cases from online clinical repositories	Multimodal (lesion photograph + incomplete descriptive text)	120 cases	GPT-4	None	Consensus seven-item structured reports from 2 oral physicians, with third-physician adjudication
Pradhan et al. [34]	2025	India	Comparative diagnostic accuracy	Oral potentially malignant disorders and early oral cancer	Most-likely diagnosis	Peer-reviewed published clinical case reports with paired lesion images	Text-only (primary) and multimodal (image + text) in secondary analysis	42 cases	ChatGPT-3.5, ChatGPT-4.0, ChatGPT-4o, Gemini	Human experts (subject-matter experts, *n* = 2)	Specialist-confirmed final diagnosis
Grinberg et al. [35]	2025	Israel	Retrospective clinical validation	Oral mucosal lesions	Differential diagnosis	Biopsy-proven lesion case descriptions from a tertiary oral-medicine center	Text-only case descriptions	98 cases analyzed (100 collected; 2 excluded)	ChatGPT-4	Human expert (oral-medicine specialist, *n* = 1)	Final histopathologic diagnosis
Cuevas-Núñez et al. [36]	2025	Spain	Retrospective comparative diagnostic performance	Oral and maxillofacial histopathology	Single diagnosis from histopathological descriptions	Retrospectively retrieved hospital histopathological descriptions originally written by board-certified pathologists	Text-only histopathological descriptions with age, sex and lesion site	102 cases	ChatGPT-4.0	None; three-pathologist panel established the reference	Original diagnoses and histopathologic descriptions reviewed to consensus by 3 board-certified pathologists
Ali et al. [37]	2025	Saudi Arabia	Comparative Diagnostic performance	Oral mucosal lesions	Final diagnosis + differential diagnosis	Clinical case scenarios collected from an Oral Medicine and Oral Pathology Division; 50 retained after external review	Text-only written clinical case scenarios	50 cases	ChatGPT-4, Microsoft Copilot	Human experts (oral-medicine consultants, *n* = 3)	Final diagnosis established from laboratory investigations, radiographs and histopathology
Suárez et al. [38]	2025	Spain	Diagnostic accuracy and repeatability	Oral mucosal lesions	Most-probable diagnosis	30 photographs randomly selected from a 60-image clinical oral/labial mucosal lesion dataset	Image-only (clinical intraoral photographs)	30 images	ChatGPT-4o	None	Independent scoring by 2 oral pathology experts using predefined textbook criteria; third-expert arbitration
Hassanein et al. [39]	2025	Egypt	Comparative diagnostic performance	Mixed oral lesions (benign, premalignant, malignant, inflammatory, reactive, infectious)	Differential diagnosis	Published multimodal clinical vignettes (text + intraoral photos + radiographs)	Multimodal (text + photos + radiographs): DeepSeek-3 text-only, GPT-4o full multimodal	80 cases	ChatGPT-4o, DeepSeek-3	Human experts (oral-medicine specialists, *n* = 4)	Final diagnoses from published case reports
Vaira et al. [40]	2025	Italy	Comparative diagnostic performance	Oral mucosal lesions and healthy oral mucosa	Single diagnosis + benign/malignant classification	Public-domain oral lesion images curated from Google Images	Image-only (public-domain oral lesion images)	110 images	ChatGPT-4o	None; five-expert panel established the reference	Five-expert panel labels based on histopathology or established clinical criteria
Tassoker [41]	2025	Türkiye	Retrospective diagnostic accuracy	Challenging oral and maxillofacial pathologies	Single most-likely diagnosis	123 cases from the Clinicopathologic Conference section of Oral Surgery, Oral Medicine, Oral Pathology, Oral Radiology (2020–2024)	Text-only narrative case descriptions (clinical, radiographic±histopathologic findings)	123 cases	ChatGPT-4o	None	Expert-confirmed final diagnosis from the original case report
Rewthamrongsris et al. [42]	2025	Thailand	Comparative diagnostic performance	OLP vs. non-OLP mucosal lesions	OLP/non-OLP classification + differential-diagnosis ranking	Histopathology-confirmed intraoral photographs	Image-only (cropped histopathology-confirmed intraoral photographs)	1,142 images	Gemini 1.5 Pro, Gemini 1.5 Flash, Claude 3 Opus, Claude 3.5 Sonnet, GPT-4o, GPT-4o mini, Llama-3.2 90B	3 CNNs on a 110-image classification subset; none for the ranking task	1,142 histopathologically confirmed OLP/non-OLP/normal-mucosa photographs
Kumar et al. [43]	2025	India	Diagnostic performance	Buccal mucosal lesion screening for oral potentially malignant disorders/early oral cancer	Binary lesion/normal classification	Standardized intraoral photographs from an oral potentially malignant disorder/early oral cancer screening cohort	Image-only with prompting variants (few-shot, RAG, RAG+CoT)	250 images from 125 patients; 125-image test set for RAG/RAG+CoT and 10-image subset for few-shot prompting	GPT-4o	Within-model prompting strategies (few-shot, RAG, RAG+CoT)	Three-specialist consensus annotations, with senior-expert verification using clinical records or histopathology
Suárez et al. [44]	2025	Spain	Retrospective exploratory diagnostic validation	Oral squamous cell carcinoma, leukoplakia and OLP	One-vs-rest detection/classification (OSCC, leukoplakia, OLP)	23 images selected from a 30-image teaching/private oral pathology repository by 2 general dentists for suspected OSCC, leukoplakia or OLP	Image-only (clinical lesion photographs)	23 images	ChatGPT-4o	None	Specialist clinicopathological diagnosis, with histopathology when available
Hassanein et al. [45]	2025	Egypt	Prospective diagnostic accuracy	Mixed oral lesions (benign, malignant, inflammatory, reactive, infectious)	Differential diagnosis	Clinic cases with standardized intraoral photos and brief clinical vignettes from tertiary oral-medicine clinics	Multimodal (intraoral photographs + brief clinical vignettes)	300 cases	Gemini Pro 2.5	Human experts (oral-medicine specialists, *n* = 2)	Histopathology-verified final diagnosis

AI, artificial intelligence; CNN, convolutional neural network; COT, chain-of-thought; GPT, generative pre-trained transformer; ICD-11, International Classification of Diseases, 11th revision; LLM, large language model; RAG, retrieval-augmented generation; SE, subject expert.

Substantial methodological heterogeneity was observed across studies. Diagnostic tasks comprised single-diagnosis identification [31, 34, 36–38, 40, 41], differential-diagnosis generation or ranking [30, 35, 37, 39, 42, 45], binary or multiclass lesion classification/detection [32, 40, 42–44], and structured diagnostic report generation [33]. Ali et al. [37], Vaira et al. [40], and Rewthamrongsris et al. [42] evaluated more than one diagnostic task and were therefore considered separately within each applicable task group. Input modality differed, with 8 studies using text-only prompting [30, 32, 34–37, 39, 41], 7 employing image-only prompting [31, 32, 38, 40, 42–44], and 5 evaluating multimodal inputs combining text with images and/or radiographs [32–34, 39, 45]. As several studies evaluated more than one prompting modality within the same investigation, modality-based study counts were not mutually exclusive. Outcome measures differed according to the task evaluated: exact-match accuracy was used for single-diagnosis identification; Top-1 ranking accuracy, Top-k inclusion, and other ranking measures were used for differential diagnosis; accuracy, sensitivity, specificity, F1-score and AUC were used for classification or detection; and item-level accuracy and recall were used for structured report generation. Agreement and repeatability measures were reported separately and were not interpreted as diagnostic correctness. Reference standards ranged from histopathologic confirmation to expert clinical consensus and validated case-report diagnoses. Given these differences, results are presented below according to diagnostic task. Input modality and lesion category are reported within each task-specific evidence group.

### Evidence Synthesized by Diagnostic Task

Table 2 summarizes the distinct diagnostic tasks evaluated across the included studies, together with their input modalities, target outputs, reference standards or comparators, task-appropriate outcome measures, and principal findings [30–45].

**Table 2.** Task-Specific Synthesis of Large Language Model Evaluations in OMFP

Study	Diagnostic task and input	Reference standard and comparator	Task-specific outcome and interpretation
Single-diagnosis identification			
Yu et al. [31]	**Target:** OLP recognition **Input/model:** Clinical photographs; ChatGPT-4o, ChatGPT (Chat-Diagrams plug-in) and Claude Opus; before/after provision of OLP diagnostic features	**Standard:** 128 confirmed OLP cases reviewed by 3 oral-mucosal specialists **Comparator:** None; specialists established the reference	**Outcome:** Recognition before→after OLP feature instruction: ChatGPT-4o 76/128 (59%)→99/128 (77%); ChatGPT (Chat-Diagrams plug-in) 87/128 (68%)→102/128 (80%); Claude Opus 19/128 (15%)→64/128 (50%) **Interpretation:** All cases were OLP; specificity against non-OLP lesions was not estimable
Pradhan et al. [34]	**Target:** Most-likely OPML/early oral-cancer diagnosis **Input/model:** Text alone or text + image; GPT models and Gemini	**Standard:** Confirmed source-case diagnosis **Comparator:** 2 subject experts	**Outcome:** Exact diagnosis: GPT-4o 27/42 (64.2%) text, 28/42 (66.6%) multimodal; Gemini 19/42 (45.2%) multimodal **Interpretation:** Expert agreement was distinct from correctness against the source-case diagnosis
Cuevas-Nunez et al. [36]	**Target:** Single diagnosis from written histopathology descriptions **Input/model:** Text; ChatGPT-4	**Standard:** Original diagnoses reviewed by consensus among 3 board-certified pathologists for 102 cases **Comparator:** None; the pathologist panel established the reference	**Outcome:** Exact 59.8%; clinically similar 9.8%; different 30.4% **Interpretation:** Clinically similar responses were not counted as exact diagnoses
Ali et al. [37]	**Target:** Final diagnosis **Input/model:** Text; ChatGPT and Copilot	**Standard:** Final diagnosis based on clinical, laboratory, radiographic and histopathological data **Comparator:** 3 oral-medicine consultants	**Outcome:** Exact diagnosis: ChatGPT 70%; Copilot 46%; consultants 64%–80% **Interpretation:** Final-diagnosis arm; not combined with the differential-list arm
Suárez et al. [38]	**Target:** Most-probable diagnosis **Input/model:** Clinical photographs only; repeated ChatGPT-4o assessments	**Standard:** Independent scoring by 2 experts using predefined oral-pathology textbook criteria; third-expert arbitration **Comparator:** None	**Outcome:** Correct-response rate for diagnosis: 58.2% **Interpretation:** No independent histopathological reference; repeatability was ancillary, not diagnostic correctness
Tassoker [41]	**Target:** Single most-likely diagnosis from integrated case descriptions **Input/model:** Text; ChatGPT-4o	**Standard:** Expert-confirmed diagnosis in 123 published cases **Comparator:** None	**Outcome:** Exact 96/123 (78.05%); clinically reasonable non-match 27/123 (21.95%) **Interpretation:** Reasonable alternatives were not treated as exact diagnoses
Vaira et al. [40]	**Target:** Single most-probable diagnosis **Input/model:** Curated clinical images; ChatGPT-4o	**Standard:** Unanimous consensus of a five-expert panel **Comparator:** None; the panel supplied the reference	**Outcome:** Exact diagnosis: 85/100 pathological cases (85%) **Interpretation:** Separate from the study’s benign/malignant classification arm
Differential-diagnosis generation and ranking			
Tomo et al. [30]	**Target:** 3 ranked differential diagnoses **Input/model:** Text case descriptions; ChatGPT-3.5 and ChatGPT-4	**Standard:** Diagnoses **confirmed** clinically, radiographically and/or by biopsy **Comparator:** Specialists, general dentists and students	**Outcome:** Correct diagnosis anywhere: ChatGPT-4 80.18%, specialists 86.64%; Top-1: 61.80% vs. 82.28% **Interpretation:** Top-k inclusion was not equivalent to exact Top-1 diagnosis
Grinberg et al. [35]	**Target:** 3 differential diagnoses **Input/model:** Text; ChatGPT-4	**Standard:** Final histopathological diagnosis **Comparator:** One oral-medicine specialist	**Outcome:** Histopathology match: ChatGPT 57%; specialist 70% **Interpretation:** Differential-list overlap was a concordance measure, not exact diagnosis
Ali et al. [37]	**Target:** Differential-**diagnosis** list **Input/model:** Text; ChatGPT and Copilot	**Standard: Prespecified** case differential/final diagnosis **Comparator:** 3 oral-medicine consultants	**Outcome:** All listed diagnoses correct: ChatGPT 74%; Copilot 60%; consultants 54%–72% **Interpretation:** Differential-list arm; not combined with final-diagnosis accuracy
Hassanein et al. [39]	**Target:** Top-1, Top-3 and Top-5 ranked differential diagnosis **Input/model:** Multimodal ChatGPT-4o and text-based DeepSeek-3	**Standard:** Final diagnoses in 80 published cases **Comparator:** 4 board-certified specialists	**Outcome:** Specialists had the highest numerical Top-1 accuracy, with no significant overall difference (*p* = 0.073); DeepSeek-3 outperformed ChatGPT-4o at Top-3 (adjusted *p* = 0.0153); no significant Top-5 difference **Interpretation:** Only the Top-3 DeepSeek-3 versus ChatGPT-4o comparison was significant; Top-k inclusion was not exact Top-1 diagnosis
Rewthamrongsris et al. [42]	**Target:** 3 ranked differentials for OLP/non-OLP photographs **Input/model:** Images; 7 multimodal LLMs	**Standard:** 1,142 histopathologically confirmed photographs **Comparator:** None for the ranking task	**Outcome:** Highest DCG: Claude 3.5 Sonnet, 0.63 **Interpretation:** Ranking quality; not exact diagnostic accuracy
Hassanein et al. [45]	**Target:** 3-item differential with probabilities **Input/model:** Text + photographs; Gemini 2.5 Pro with direct, COT and self-reflection prompting	**Standard:** Histopathological diagnoses for 300 cases from 2 oral pathologists **Comparator:** 2 oral-medicine specialists	**Outcome:** Top-1: experts 61%, COT 55%; Top-3: COT 82%, experts 76%; overall *p* = 0.41 and *p* = 0.39, respectively **Interpretation:** Numerical differences only; neither overall comparison was statistically significant
Binary or multiclass lesion classification/detection			
Schmidl et al. [32]	**Target:** SCC/leukoplakia/no-lesion 3-class classification **Input/model:** Image, history or combined input; ChatGPT-4	**Standard:** Pathological cases histologically confirmed; no-lesion images served as controls **Comparator:** None	**Outcome:** SCC sensitivity/specificity/accuracy: image 18.2%/52.2%/35.6%; combined 100%/88.2%/91.1% **Interpretation:** Lesion-specific classification; not general OMFP diagnostic accuracy
Vaira et al. [40]	**Target:** Benign/malignant classification **Input/model:** Clinical images; ChatGPT-4o	**Standard:** Expert-panel labels based on histopathology or well-established clinical criteria **Comparator:** None; panel supplied the labels	**Outcome:** Sensitivity 91.7%; specificity 100%; accuracy 97.0%; 3 malignant lesions missed **Interpretation:** No independent expert-performance comparison
Rewthamrongsris et al. [42]	**Target:** OLP/non-OLP classification **Input/model:** Images; 7 LLMs with zero-shot or example-guided prompting	**Standard:** 1,142 histopathologically confirmed photographs **Comparator:** 3 CNNs on a 110-image subset	**Outcome:** Zero-shot maxima: accuracy 69.69% (Gemini 1.5 Pro/Flash) and F1 76.10% (GPT-4o); example-guided maxima: accuracy 80.53% and F1 84.54% (Gemini 1.5 Flash); all 3 CNNs outperformed the LLMs on the subset **Interpretation:** Classification metrics; separate from the study’s ranking task
Kumar et al. [43]	**Target:** Lesion/normal classification **Input/model:** Smartphone images; GPT-4o with few-shot, RAG or RAG + COT	**Standard:** Three-specialist annotations with consensus, followed by senior-expert verification using clinical records or histopathology **Comparator:** None	**Outcome:** Sensitivity/specificity/accuracy/F1: few-shot 80%/100%/90%/0.88 (*n* = 10); RAG 54%/91%/68%/0.68; RAG+COT 90%/64%/82%/0.86 **Interpretation:** Unequal test-set sizes, including *n* = 10 for few-shot, precluded direct comparison across strategies
Suárez et al. [44]	**Target:** One-vs-rest OSCC, leukoplakia and OLP detection **Input/model:** Clinical images; repeated ChatGPT-4o assessments	**Standard:** Specialist clinicopathological diagnosis, with histopathology when available **Comparator:** None	**Outcome:** Sensitivity/specificity/AUC: OSCC 65%/97.14%/0.81; leukoplakia 60%/100%/0.80; OLP 25%/96.11%/0.61 **Interpretation:** Entity-specific detection; not exact diagnosis
Structured diagnostic report generation			
Zhan et al. [33]	**Target:** Seven-item structured diagnostic report **Input/model:** Lesion photograph + incomplete text; GPT-4	**Standard:** Consensus reports from 2 oral physicians, with third-physician adjudication **Comparator:** None	**Outcome:** Textbook/internet-source accuracy 87.05%/84.87%; recall 90.73%/89.12%; diagnosis-item accuracy 91.11% **Interpretation:** Item-level report measures; not whole-case exact diagnosis

Multi-task studies are shown in each applicable task group. Exact-match, Top-k, classification and report-generation outcomes were interpreted only within their respective tasks; agreement, plausibility, calibration and repeatability were treated as ancillary outcomes rather than interchangeable measures of diagnostic correctness.

AUC, area under the receiver-operating-characteristic curve; CNN, convolutional neural network; COT, chain-of-thought; DCG, discounted cumulative gain; F1, F1-score; LLM, large language model; OLP, oral lichen planus; OMFP, oral and maxillofacial pathology; OPML, oral potentially malignant lesion; OSCC, oral squamous cell carcinoma; RAG, retrieval-augmented generation; SCC, squamous cell carcinoma.

### Single-Diagnosis Identification

Seven studies evaluated a single most-likely or final diagnosis, or recognition of a prespecified lesion [31, 34, 36–38, 40, 41]. These studies differed substantially in case spectrum and reference standard. Yu et al. [31] evaluated recognition of OLP within a dataset containing only confirmed OLP cases; consequently, the reported improvements following lesion-specific instruction demonstrated recognition within that cohort but did not establish specificity against non-oral-lichen-planus lesions. Pradhan et al. [34] reported exact-diagnosis accuracy of 64.2% for GPT-4o with text-only cases and 66.6% with multimodal cases, whereas Gemini achieved 45.2% in the multimodal arm. Cuevas-Nunez et al. [36] reported 59.8% exact agreement with the original pathologist diagnosis; clinically similar but non-identical diagnoses were reported separately and were not counted as exact matches.

In Ali et al. [37], final-diagnosis accuracy was 70% for ChatGPT and 46% for Copilot, compared with 64%–80% among the 3 oral-medicine consultants. Suarez et al. [38] reported a 58.2% correct-response rate for image-based probable diagnosis, but responses were assessed against predefined expert-scored criteria rather than an independent histopathological reference standard. Tassoker [41] reported 78.05% exact diagnostic accuracy; the remaining clinically reasonable differentials were not considered correct final diagnoses. Vaira et al. [40] reported 85% agreement with expert-consensus labels, but the expert panel provided the reference labels rather than acting as an independent performance comparator.

Accordingly, the single-diagnosis evidence demonstrates variable exact-label performance across heterogeneous datasets and reference standards. It does not establish consistent equivalence between LLMs and specialists.

### Differential-Diagnosis Generation and Ranking

Six studies evaluated the generation or ranking of differential diagnoses [30, 35, 37, 39, 42, 45]. Tomo et al. [30] reported inclusion of a correct diagnosis within the generated differential in 80.18% of ChatGPT-4 outputs and 86.64% of specialist outputs, whereas the correct diagnosis was ranked first in 61.80% and 82.28%, respectively. These outcomes demonstrate the distinction between inclusion within a differential and selection as the primary diagnosis. Grinberg et al. [35] assessed overlap between ChatGPT and a specialist differential, as well as correspondence with histopathology; differential overlap measured concordance rather than diagnostic correctness. Ali et al. [37] reported study-specific differential-diagnosis scores separately from the study’s final-diagnosis arm.

In Hassanein et al. [39], specialists achieved the highest overall Top-1 performance, whereas DeepSeek-3 exceeded ChatGPT-4o at Top-3; no significant model difference was observed at Top-5. Rewthamrongsris et al. [42] evaluated differential-ranking quality using first-ranked, any-of-three, and discounted cumulative gain measures, with Claude 3.5 Sonnet achieving the highest discounted cumulative gain. Hassanein et al. [45] found that specialists achieved higher Top-1 accuracy, whereas chain-of-thought prompting produced higher Top-3 inclusion than the specialists within that study.

These findings indicate that LLMs may assist in broadening or ranking differential diagnoses. However, Top-3 or Top-5 inclusion represents retrieval of the reference diagnosis within a candidate list and should not be interpreted as equivalent to an exact correct diagnosis.

### Binary or Multiclass Lesion Classification and Detection

Five studies evaluated defined lesion classification or detection tasks [32, 40, 42–44]. Schmidl et al. [32] classified cases as squamous cell carcinoma, leukoplakia, or no lesion and demonstrated substantial variation by input condition: for squamous cell carcinoma, sensitivity, specificity and accuracy were 18.2%, 52.2%, and 35.6% with image-only input, compared with 100%, 88.2%, and 91.1% when image and clinical history were combined. Vaira et al. [40] separately evaluated benign-versus-malignant classification, reporting sensitivity of 91.7% and specificity of 100%; this binary task was distinct from the study’s exact-diagnosis assessment.

Rewthamrongsris et al. [42] evaluated oral-lichen-planus versus non-oral-lichen-planus classification separately from differential ranking. Example-guided prompting improved LLM classification, although all three CNN comparators outperformed the LLMs. Kumar et al. [43] evaluated lesion-versus-normal screening and reported varying sensitivity, specificity, accuracy and F1-score across few-shot and retrieval-augmented prompting strategies; the small test subsets limited direct comparison. Suarez et al. [44] performed separate one-versus-rest detection of oral squamous cell carcinoma, leukoplakia and OLP, with relatively high specificity but markedly variable sensitivity across the 3 target conditions.

The classification evidence therefore suggests that LLMs may achieve high specificity in some narrowly defined tasks, but sensitivity remains variable and task-dependent. These results cannot be generalized to exact diagnosis across the broader OMFP spectrum.

### Structured Diagnostic Report Generation

One study evaluated structured diagnostic report generation [33]. Zhan et al. assessed completion of 7 report components, including lesion location, shape, number, size, clinical manifestation, border and diagnosis. Overall report accuracy was 87.05% for the textbook dataset and 84.87% for the internet dataset, with corresponding recall values of 90.73% and 89.12%. These were item-level structured-report measures; although the diagnosis component achieved 91.11% accuracy, the overall report results should not be interpreted as whole-case exact diagnostic accuracy. Because this task was evaluated by only 1 study, no cross-study conclusion regarding report generation can currently be drawn.

### Secondary Influence of Input Modality

Input modality was examined as a secondary influence within comparable diagnostic tasks. Within-study comparisons suggested that adding clinical history to images improved classification performance in Schmidl et al. [32] and produced a smaller improvement in single-diagnosis accuracy in Pradhan et al. [34]. However, comparisons between text-only, image-only and multimodal results from different studies were not treated as direct modality comparisons because the diagnostic tasks, datasets, reference standards and outcome measures differed.

### Secondary Interpretation by Lesion Category

Lesion category was interpreted as a secondary source of heterogeneity within diagnostic-task groups (Table 3). For inflammatory and autoimmune oral mucosal lesions, particularly OLP, studies evaluated nonequivalent tasks including recognition within an all-OLP cohort, OLP/non-OLP classification, differential-diagnosis ranking, and one-vs-rest detection. Accordingly, no single lesion-level accuracy estimate was inferred. In the dedicated classification comparison, CNNs outperformed the evaluated LLMs, while another small image-based study reported low OLP sensitivity [31, 42, 44].

**Table 3.** Lesion-Category-Stratified Secondary Synthesis of Large Language Model Evaluations Across Diagnostic Tasks in OMFP

Lesion category	Included studies	Representative lesions	Main findings	Interpretation
Inflammatory and autoimmune oral mucosal lesions	Yu et al. [31], Grinberg et al. [35], Cuevas-Núñez et al. [36], Hassanein et al. [39], Tassoker [41], Rewthamrongsris et al. [42], Suárez et al. [44], Hassanein et al. [45]	OLP, lichenoid lesions, mucous membrane pemphigoid, lupus-associated bullous lesions, granulomatous inflammatory lesions	Performance varied across nonequivalent OLP tasks, including all-OLP recognition, OLP/non-OLP classification and one-vs-rest detection. In the classification study, CNNs outperformed the evaluated LLMs on the tested subset; image-based OLP sensitivity was low in another small study	OLP-focused evidence showed variable image-based performance across the evaluated tasks, but task heterogeneity prevents a single lesion-level accuracy estimate; specialist interpretation and histopathologic correlation remain important when clinically indicated
Oral potentially malignant disorders and malignant epithelial lesions	Schmidl et al. [32], Pradhan et al. [34], Grinberg et al. [35], Cuevas-Núñez et al. [36], Hassanein et al. [39], Vaira et al. [40], Tassoker [41], Kumar et al. [43], Suárez et al. [44], Hassanein et al. [45]	Leukoplakia, erythroplakia, epithelial dysplasia, oral squamous cell carcinoma, verrucous carcinoma, carcinoma cuniculatum	Results varied by task and input. Adding clinical history markedly improved SCC classification in Schmidl et al.; Vaira et al. reported high benign/malignant classification performance but missed 3 malignant lesions; Suárez et al. [44] showed high specificity but only 65% OSCC and 60% leukoplakia sensitivity. Exact-diagnosis and Top-k outcomes were not directly comparable with these classification results	These studies support further evaluation for adjunctive triage and screening, but false negatives and task heterogeneity preclude autonomous diagnostic use
Reactive, infectious, and benign oral mucosal lesions	Zhan et al. [33], Grinberg et al. [35], Cuevas-Núñez et al. [36], Ali et al. [37], Suárez et al. [38], Hassanein et al. [39], Vaira et al. [40], Tassoker [41], Hassanein et al. [45]	Mucocele, fibroma, pyogenic granuloma, candidiasis, traumatic ulcerative lesions, reactive lesions	These entities were represented mainly within heterogeneous oral mucosal lesion datasets. Outcomes spanned exact diagnosis, differential-list performance, image classification and structured-report generation, so lesion-specific performance could not be compared directly across studies	Evidence remains concentrated in oral-medicine-oriented mucosal workflows, with insufficient task-consistent data for firm conclusions about reactive, infectious or benign subgroups
Salivary gland lesions	Tomo et al. [30], Tassoker [41]	Mucoepidermoid carcinoma, adenoid cystic carcinoma, secretory carcinoma, basal cell adenoma	Salivary gland lesions were represented only incidentally within mixed OMFP datasets	No dedicated salivary gland-specific validation study was identified among the included studies
Odontogenic and osseous lesions	Tomo et al. [30], Cuevas-Núñez et al. [36], Tassoker [41]	Dentigerous cyst, ameloblastoma, odontogenic keratocyst, ossifying fibroma, aneurysmal bone cyst	Odontogenic and osseous lesions were represented only within mixed case collections, with limited lesion-specific outcome reporting	Current evidence is insufficient for lesion-specific conclusions
Mesenchymal, vascular and soft-tissue lesions	Cuevas-Núñez et al. [36], Tassoker [41]	Hemangioma, lymphangioma, myofibroma, synovial sarcoma, leiomyosarcoma, lipoma, fibroma	These lesions were only sporadically represented within mixed OMFP or histopathology-description datasets	Current evidence does not permit reliable conclusions for mesenchymal or soft-tissue lesion diagnosis
Mixed OMFP entities	Tomo et al. [30], Cuevas-Núñez et al. [36], Hassanein et al. [39], Tassoker [41], Hassanein et al. [45]	Mixed inflammatory, benign, malignant, salivary, odontogenic, osseous and mesenchymal lesions	Mixed datasets showed variable performance across exact-diagnosis and Top-k differential tasks. In Hassanein et al. [45], chain-of-thought prompting produced the highest numerical Top-3 accuracy (82%), but the overall comparison with experts was not statistically significant	Evidence remains preliminary and heterogeneous; results from mixed datasets should not be generalized as lesion-specific diagnostic accuracy

CNN, convolutional neural network; LLM, large language model; OLP, oral lichen planus; OSCC, oral squamous cell carcinoma; SCC, squamous cell carcinoma. Top-k, correct diagnosis appearing within the first *k* ranked diagnostic outputs.

For oral potentially malignant disorders and malignant epithelial lesions, findings similarly depended on the diagnostic task and input. Adding clinical history markedly improved squamous cell carcinoma classification in Schmidl et al. [32]; Vaira et al. [40] reported high benign/malignant classification performance but missed 3 malignant lesions; and Suárez et al. [44] reported high specificity with sensitivities of 65% for oral squamous cell carcinoma and 60% for leukoplakia. Exact-diagnosis and Top-k differential-diagnosis outcomes were not compared directly with these classification results.

Reactive, infectious, and benign oral mucosal lesions were predominantly represented within heterogeneous datasets using different diagnostic tasks, including exact diagnosis, differential-diagnosis generation, classification, and structured-report generation. Consequently, task-consistent lesion-specific conclusions could not be drawn. Salivary gland, odontogenic, osseous, mesenchymal, vascular, and soft-tissue lesions were represented only sporadically within mixed datasets, and the available evidence is insufficient for lesion-specific conclusions [30, 33, 35–41, 45]. Overall, lesion category modified performance within individual diagnostic tasks, but differences between lesion categories should not be interpreted independently of the task, input modality, reference standard, and outcome measure used.

Within the individual diagnostic-task groups, outcomes varied according to lesion category, dataset composition, case complexity, and input modality. Evidence was concentrated predominantly on oral mucosal and oral-medicine-related conditions, whereas odontogenic, salivary gland, mesenchymal, and osseous lesions were sparsely represented. Because different lesion categories were assessed using different diagnostic tasks and outcome measures, no overall lesion-spanning estimate of LLM diagnostic performance was inferred.

### Risk-of-Bias Assessment

QUADAS-2 assessment demonstrated a heterogeneous risk-of-bias profile across the included studies, with recurrent methodological limitations identified across multiple domains (Table 4). Patient selection represented the principal source of bias, with 15 of the 16 studies rated as high risk because of retrospective convenience sampling, restricted diagnostic cohorts, or exclusion of ambiguous cases, thereby limiting representativeness and increasing susceptibility to spectrum bias [30–44]. Only the prospective multicenter study by Hassanein et al. [45] received a low-risk rating because of its histopathology-verified recruitment methodology.

**Table 4.** QUADAS-2 Risk of Bias and Applicability Concerns of Included Studies

Study	Patient selection	Index test	Reference standard	Flow and timing	Applicability: patient selection	Applicability: index test	Applicability: reference standard
Tomo et al. [30]	High	Low	Low	Low	High	Low	Low
Yu et al. [31]	High	High	Low	Low	High	High	Low
Schmidl et al. [32]	High	Unclear	Low	Low	High	Unclear	Low
Zhan et al. [33]	High	Unclear	Low	Low	High	Unclear	Low
Pradhan et al. [34]	High	High	Low	Unclear	High	High	Low
Grinberg et al. [35]	High	Unclear	Low	Low	High	Unclear	Low
Cuevas-Núñez et al. [36]	High	Low	Low	Low	High	Unclear	Low
Ali et al. [37]	High	Unclear	Low	Low	High	Unclear	Low
Suárez et al. [38]	High	Unclear	Low	Low	High	Unclear	Low
Hassanein et al. [39]	High	Unclear	Low	Low	High	Unclear	Low
Vaira et al. [40]	High	Unclear	Low	Low	High	Unclear	Low
Tassoker [41]	High	Unclear	Low	Low	High	Unclear	Low
Rewthamrongsris et al. [42]	High	Unclear	Low	Low	High	Unclear	Low
Kumar et al. [43]	High	Unclear	Unclear	High	Unclear	Low	Unclear
Suárez et al. [44]	High	Unclear	Low	Low	High	Unclear	Low
Hassanein et al. [45]	Low	Low	Low	Low	Low	Low	Low

In the Index Test domain, 11 studies were rated as unclear risk because of insufficient reporting of prompting strategies, temperature settings, blinding procedures, and measures to reduce test-retest variability [32, 33, 35, 37–44]. Two studies [31, 34] were classified as high risk because their prompting structures incorporated disease-specific cues potentially aligned with the reference standard. In contrast, Tomo et al. [30], Cuevas-Núñez et al. [36], and Hassanein et al. [45] demonstrated reproducible and independently conducted index test procedures and were therefore rated as low risk. The Reference Standard domain demonstrated the greatest methodological consistency, with 15 studies rated as low risk because of the use of expert consensus or histopathological confirmation [30–42, 44, 45]. Kumar et al. [43] received an unclear risk rating because of insufficient detail regarding the adjudication process, whereas no study was classified as high risk in this domain. The Flow and Timing domain demonstrated the lowest overall risk of bias, with 14 studies rated as low risk because eligible cases were consistently evaluated using both the index test and the reference standard [30–33, 35–42, 44, 45]. Pradhan et al. [34] received an unclear rating because of insufficient reporting regarding case exclusion handling, whereas Kumar et al. [43] was rated as high risk because of documented case attrition between LLM assessment and reference standard evaluation.

Regarding applicability, the greatest concerns were observed in the Patient Selection domain. Fourteen studies demonstrated high applicability concern because they relied on curated datasets, specialist-level imagery, or highly structured case descriptions rather than representative clinical case mixes [30–42, 44]. Kumar et al. [43] were rated as unclear because of insufficient reporting regarding dataset representativeness, whereas Hassanein et al. [45] demonstrated low applicability concern across all domains. Applicability concerns related to the Index Test domain were frequently rated as unclear because of variability in prompting strategies, multimodal input handling, and evaluation pipelines [32, 33, 35–42, 44]. Two studies [31, 34] demonstrated high applicability concern because their prompting approaches may not reflect real-world clinical implementation. Tomo et al. [30], Kumar et al. [43], and Hassanein et al. [45] demonstrated low applicability concern in this domain. In contrast, applicability concerns related to the Reference Standard domain were low in 15 studies because diagnostic benchmarks were aligned with accepted clinical practice [30–42, 44, 45], whereas Kumar et al. [43] remained unclear.

Overall, methodological concerns were primarily related to patient selection and index test implementation, reflecting retrospective sampling strategies, curated datasets, and insufficient reporting of prompting methodologies. In contrast, the Reference Standard and Flow and Timing domains generally demonstrated low risk of bias and low applicability concern across most included studies (Fig. 2).

**Fig. 2.**
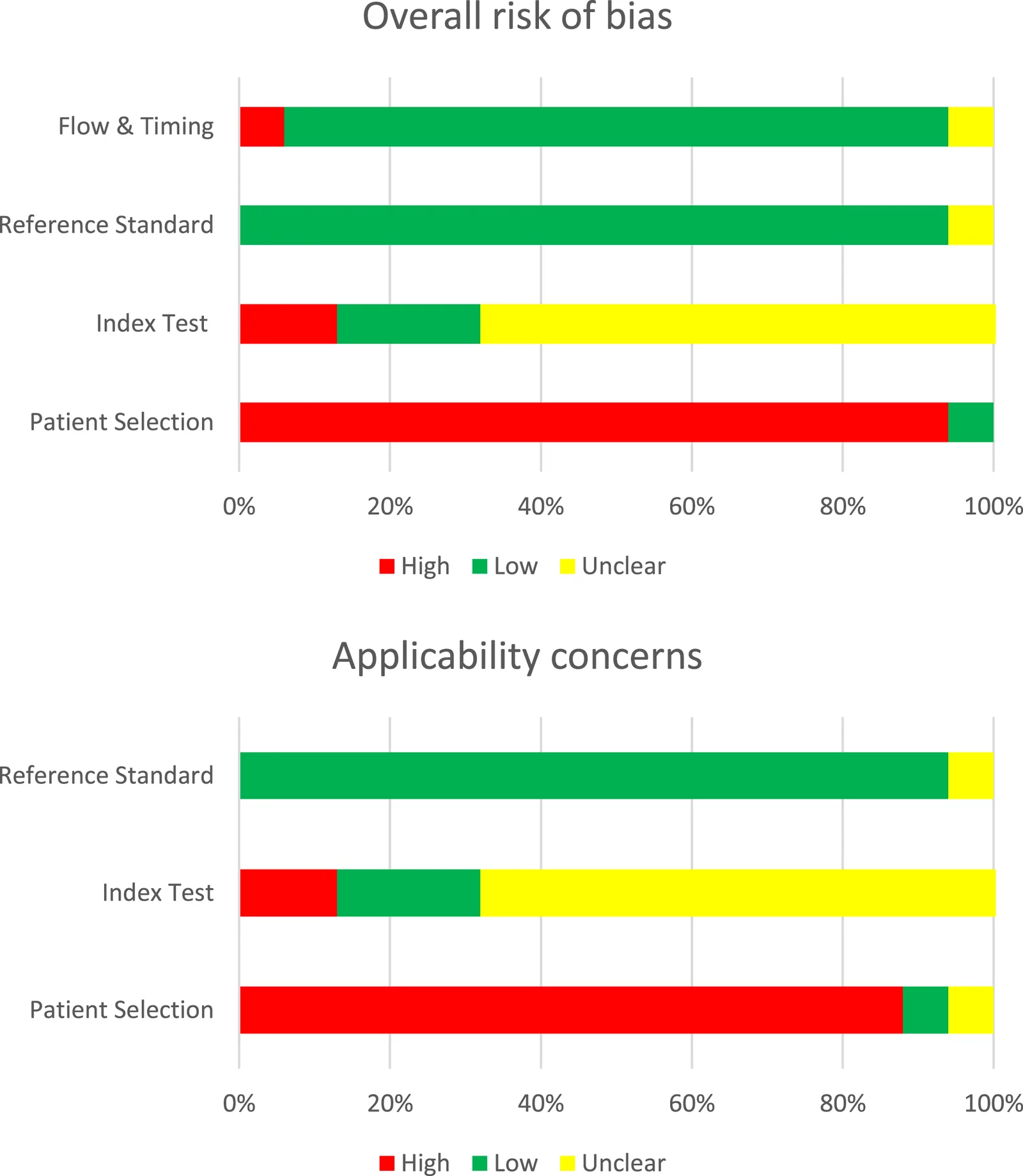
QUADAS-2 assessment of risk of bias (upper panel) and applicability concerns (lower panel) across the 16 included studies. Red indicates high risk of bias or high applicability concerns, green indicates low risk or low concerns, and yellow indicates unclear assessments.

## Discussion

This systematic review demonstrates that the available evidence does not represent a single, interchangeable construct of LLM diagnostic performance. The included studies evaluated materially different tasks: single-diagnosis identification, differential-diagnosis generation or ranking, binary or multiclass lesion classification/detection, and structured diagnostic report generation [30–45]. Consequently, conclusions should be confined to studies evaluating comparable tasks using appropriate outcome measures and reference standards.

In single-diagnosis studies, LLM performance varied substantially across datasets, lesion spectra, input conditions, and reference standards [31, 34, 36–38, 40, 41]. Among single-diagnosis studies with independent human comparators, findings were mixed and did not establish consistent specialist equivalence [34, 37]. In Vaira et al. [40], the expert panel established the reference diagnosis and therefore did not constitute an independent performance comparator. Agreement with clinicians, correspondence with expert-consensus labels, or production of clinically plausible alternatives should not be interpreted as equivalent to correctness against an independent reference diagnosis [34–36, 38, 40, 41].

Differential-diagnosis studies showed a different pattern. Large language models frequently included the reference diagnosis within a Top-3 or Top-5 list even when it was not selected as the primary diagnosis [30, 39, 42, 45]. This supports a possible role in expanding or ranking candidate diagnoses, but Top-k inclusion does not establish a correct final diagnosis. Statements comparing LLMs with specialists must therefore specify whether the comparison concerns Top-1 selection, Top-k inclusion, differential overlap, or ranking quality [30, 35, 39, 42, 45].

In classification and detection tasks, specificity was often high, but sensitivity varied markedly according to the target lesion, prompting strategy, and input condition [32, 40, 42–44]. Dedicated CNN systems outperformed the evaluated LLMs in the oral-lichen-planus classification study, indicating that current general-purpose LLMs should not be assumed to match specialized vision models [42]. Structured diagnostic report generation was evaluated in only 1 study, and its item-level accuracy and recall cannot be generalized to whole-case diagnostic accuracy or to report generation across OMFP [33].

Input modality and lesion category modified outcomes within these diagnostic tasks but should be regarded as secondary explanatory factors. Within-study comparisons suggested that adding clinical context to images improved classification performance in Schmidl et al. [32] and produced a smaller improvement in single-diagnosis accuracy in Pradhan et al. [34]. Nevertheless, cross-study comparisons between text-only, image-only, and multimodal performance remain unreliable when the underlying tasks, datasets, reference standards, and outcome measures differ. The evidence base was concentrated predominantly on oral mucosal and oral-medicine-related conditions [31, 32, 34, 38, 42–44], with comparatively limited representation of odontogenic, salivary gland, mesenchymal, and osseous pathology [30, 36, 41].

Broader medical and pathology literature provide contextual support for these methodological observations, although these findings were not combined with the task-specific OMFP evidence. Evaluations of GPT-4 in complex clinical cases and LLM-assisted differential diagnosis have demonstrated potential for clinician support while emphasizing the need for further real-world validation [46, 47]. Studies in general and surgical pathology likewise indicate that LLM performance varies according to the diagnostic task and input representation [48, 49], while reviews of oral pathology and medical diagnostics highlight heterogeneous performance and the need for specialist oversight and rigorous validation [50, 51]. In histopathologic image assessment, performance has also varied according to image selection and the use of in-context examples [52]. AI-based approaches have demonstrated strong performance in narrowly defined oral image-classification and detection tasks, while the broader literature also highlights the need for continued task-specific validation [53–56].

Prompt design may influence performance within individual diagnostic tasks, but the available studies do not support a general cross-task conclusion that structured prompting is superior. In Rewthamrongsris et al. [42], example-guided prompting improved OLP classification performance. In Kumar et al. [43], prompting strategies were evaluated using unequal test-set sizes, limiting direct comparison. In Hassanein et al. [45], chain-of-thought prompting produced the highest numerical Top-3 accuracy, but the overall comparison with experts was not statistically significant. Prompt effects should therefore be interpreted within the specific diagnostic task, dataset, reference standard, and outcome evaluated. Broader medical studies similarly indicate that reasoning-oriented prompting and retrieval-augmented generation can alter model performance, but the magnitude and direction of benefit are dependent on the task, data and evaluation setting [57–60].

Despite encouraging findings within some individual diagnostic tasks, the available evidence remains methodologically fragile. This heterogeneity was particularly evident across input modalities, lesion categories, prompting strategies, and reference standards. Although several studies compared more than one LLM or more than one prompting strategy, methodological heterogeneity in tasks, thresholds and outcome definitions limited cross-study comparability. Hence, meta-analysis was not possible. Most studies featured small sample sizes, retrospective designs, curated case mixes, or vignette-based inputs that do not reflect real-world case diversity. Human clinician comparators were inconsistently included, external validation was rare, and only a minority of studies assessed the impact of LLM-assisted workflows on clinical decision-making. Similar concerns have been raised in broader LLM evaluations across dentistry, highlighting that current studies are exploratory and insufficient to support autonomous deployment [25]. In addition, risks of hallucinated diagnoses, fabricated references, and confidently incorrect clinical explanations remain a major barrier to unsupervised clinical use [61]. Consequently, LLM-generated outputs must be treated as advisory and never as definitive, with final diagnosis remaining the responsibility of the clinician. Beyond technical limitations, safe deployment will require regulatory clarity around diagnostic responsibility in AI-assisted care, compliance with medical device regulations such as the US Food and Drug Administration pathway for Software as a Medical Device, and safeguards to prevent automation bias while ensuring that clinicians not algorithms remain the final decision makers.

Nonetheless, LLMs may have potential roles within selected components of the OMFP diagnostic workflow. One promising application is triage, where LLMs could help flag suspicious oral lesions for urgent biopsy or referral, a capability that would be particularly valuable in resource-limited environments with constrained specialist access. Large language models may also function as second readers, broadening differential diagnoses and counteracting cognitive biases such as anchoring and premature closure, thereby supporting safer decision-making. In addition, their capacity for structured, explanation-based reasoning positions them as effective educational tools for dental students and general practitioners, enabling case-based feedback that scaffolds diagnostic learning. Another notable application is documentation support, including the generation of structured reports and the synthesis of relevant literature to improve efficiency and clarity of clinical communication. Importantly, these capabilities should not be viewed as replacements for oral and maxillofacial pathologists but rather as augmentative tools capable of enhancing diagnostic safety, equity, and accessibility.

Looking ahead, progress in this field will require a shift from retrospective accuracy studies to prospective, multicenter clinical trials capable of measuring the real-world impact of LLM-assisted diagnostics. Equally essential is the development of large and representative multimodal datasets that capture the full lesion continuum rather than the current over-representation of some specific common lesions. The potential value of domain-specific multimodal LLMs fine-tuned for pathology and capable of integrating images, radiographs, and histology should be evaluated against general-purpose models. Task-specific benchmarking frameworks are needed to enable fair comparison across systems, with outcome measures matched to the diagnostic task evaluated; calibration and clinician-level utility should be assessed as complementary outcomes rather than substitutes for diagnostic correctness. Safety-critical safeguards must include uncertainty communication, hallucination prevention, grounding in verifiable sources, and explainable reasoning. Finally, the human-AI interface warrants dedicated investigation, where prospective studies should evaluate whether LLM support improves diagnostic confidence and accuracy or whether it introduces automation bias.

In conclusion, the available evidence evaluates several nonequivalent LLM diagnostic tasks in OMFP and does not support a single overall estimate of diagnostic performance. Exact-diagnosis performance remains inconsistent across heterogeneous datasets and reference standards. Differential-diagnosis studies suggest potential utility for generating or ranking candidate diagnoses, but Top-k inclusion should not be interpreted as correct final diagnosis. Classification and detection studies demonstrate high specificity in some settings but variable, task-dependent sensitivity, while evidence for structured diagnostic report generation is limited to a single study. Large language models should therefore be regarded as adjunctive tools, and current evidence does not support autonomous diagnostic use in OMFP.

## Statement of Ethics

This study is a systematic review of previously published literature and did not involve the collection of primary data from human participants or animals. Ethical approval was therefore not required. This exemption was determined in accordance with institutional and journal guidelines. No human participants were directly involved in this study, and informed consent was not required.

## Conflict of Interest Statement

The authors have no conflicts of interest to declare.

## Funding Sources

This study was not supported by any sponsor or funder.

## Author Contributions

Conceptualization: S.S.; methodology: S.S., M.R., H.A.; formal analysis and investigation: S.S., M.R., H.A.; writing – original draft preparation: S.S.; writing – review and editing: S.S., M.R., H.M.; resources: S.S.; supervision: S.S.

## Data Availability

All data generated or analyzed during this study are included in this published article and its online supplementary material. No additional datasets were generated during the current study. The database-specific search strategies and completed PRISMA 2020 checklist are provided as online supplementary Files 1 and 2, respectively. Further inquiries can be directed to the corresponding author.
